# The PTEN–PI3K Axis in Cancer

**DOI:** 10.3390/biom9040153

**Published:** 2019-04-17

**Authors:** Antonella Papa, Pier Paolo Pandolfi

**Affiliations:** 1 Cancer Program, Monash Biomedicine Discovery Institute and Department of Biochemistry and Molecular Biology, Monash University, Melbourne, Victoria 3800, Australia; 2 Cancer Research Institute, Beth Israel Deaconess Cancer Center, Department of Medicine and Pathology, Beth Israel Deaconess Medical Center (BIDMC), Harvard Medical School, Boston, MA 02215, USA

**Keywords:** PTEN, PI3K, cancer predisposition syndromes, targeted therapies, mouse models of human cancer

## Abstract

The PI3K–AKT–mTOR signal transduction pathway regulates a variety of biological processes including cell growth, cell cycle progression and proliferation, cellular metabolism, and cytoskeleton reorganization. Fine-tuning of the phosphatidylinositol 3-kinase (PI3K) pathway signaling output is essential for the maintenance of tissue homeostasis and uncontrolled activation of this cascade leads to a number of human pathologies including cancer. Inactivation of the tumor suppressor phosphatase and tensin homologue deleted on Chromosome 10 (PTEN) and/or activating mutations in the proto-typical lipid kinase PI3K have emerged as some of the most frequent events associated with human cancer and as a result the PI3K pathway has become a highly sought-after target for cancer therapies. In this review we summarize the essential role of the PTEN–PI3K axis in controlling cellular behaviors by modulating activation of key proto-oncogenic molecular nodes and functional targets. Further, we highlight important functional redundancies and peculiarities of these two critical enzymes that over the last few decades have become a central part of the cancer research field and have instructed hundreds of pre-clinical and clinical trials to better cancer treatments.

## 1. The PI3K Pathway: Molecular Hubs and Biological Functions

The phosphatidylinositol 3-kinase (PI3K) is the upstream lipid kinase of the PI3K–AKT–mTOR signal transduction pathway. Structural and functional specificities identify different classes of PI3Ks with class IA PI3Ks existing as heterodimers of a p85 type of regulatory subunit and a p110 multi-isoform catalytic subunit: p110α, p110β, and p110δ [1]. All p110-isoforms contribute to class IA PI3Ks signaling but the p110α-encoding gene, *PIK3CA*, is the only isoform frequently mutated in cancer [2,3].

In physiological conditions, receptor tyrosine kinases (RTKs) and G-protein coupled receptors (GPCRs) activate PI3K which then catalyzes the phosphorylation of the lipid substrate phosphatidylinositol (4,5)-bisphosphate (PIP2) to generate phosphatidylinositol (3,4,5)-trisphosphate (PIP3) [4]. PIP3 is an essential second messenger that amplifies the PI3K signal by recruiting proteins containing lipid-binding domains, such as the serine/threonine AGC kinase AKT and the phosphoinositide-dependent protein kinase-1 (PDK1). At the membrane, PDK1 directly phosphorylates and activates AKT on Thr 308 which, in turn, can engage and activate over 100 reported effector targets including the glycogen synthase kinase 3 (GSK3), the Forkhead Box O (FoxO) proteins, and the mammalian (or mechanistic) target of rapamycin complex 1 (mTORC1), thus controlling multiple pro-survival cellular processes [5,6]. Maximal AKT activation, however, requires an additional phosphorylation event on Ser 473 which is catalyzed by the mTOR complex2 (mTORC2) which can also be regulated by PIP3 [7].

The AKT control of mTORC1 functional status occurs through multiple molecular mechanisms one of which involves inhibition of the tumor suppressive tuberous sclerosis complex (TSC). Upon growth factors stimulation, AKT phosphorylation of tuberous sclerosis complex 2 (TSC2) within the TSC, releases an inhibitory switch that allows the Ras-related GTPase Rheb to be GTP loaded and to activate mTORC1 on lysosomes [8]. At the lysosomes, mTORC1 engages with members of the Ragulator complex and in presence of nutrient availability activates anabolic processes, such as lipid and nucleotide synthesis, protein translation, and ribosomal biogenesis, and sustains cell growth and proliferation [6]. Thus, by transducing signals from the upstream cytoplasmic membrane complexes to the endocellular organelles, the PI3K pathway and its molecular hubs establish an essential temporal and spatial signaling network that arms cells with the necessary biomolecules to allow cellular growth during embryonic development and in adulthood for tissue growth and regeneration.

Because of its essential role in controlling multiple pro-survival processes, activation of the PI3K signal is tightly modulated by negative regulators that act by ensuring the timely inhibition of the pathway and by preventing excessive growth. A critical upstream member of the cascade is the tumor suppressor phosphatase and tensin homolog deleted on Chromosome 10 (PTEN), a dual-specificity lipid and protein phosphatase that efficiently dephosphorylates the 3′-group of PIP3 and, therefore, terminates propagation of the signal to AKT and other PIP3-effector targets [9]. The presence and activation of PTEN at the cytoplasmic membrane are crucial to guarantee controlled transduction of the PI3K signal which is then transmitted as a healthy message to the cell. Consequently, the removal of this upstream break unleashes a continuous positive signal that translates into uncontrolled overgrowth [10]. Similarly, constitutive activation of the pathway achieved through the acquisition of oncogenic *PIK3CA* mutations overcomes the negative regulation imposed by PTEN with ensuing tumorigenesis [4]. Thus, PTEN and PI3K define a key functional axis that in a coordinated fashion modulates the activation status of multiple proto-oncogenic signals that can be scavenged during tumorigenesis and are frequently exploited by cancerous cells for survival.

## 2. The Tumors Suppressor PTEN

### 2.1. Mutations, Lipid Function and In Vivo Studies

*PTEN* is one of the most frequently mutated tumor suppressor genes in human cancer [10]. *PTEN* is expressed early during embryogenesis and ubiquitously throughout adulthood, and its functional loss can have dramatic consequences to cellular and organismal homeostasis [11,12,13]. *PTEN* mutations occur in somatic and hereditary tumor syndromes, and both conditions lead to cellular overgrowth with potential cancer progression overtime [14]. Germline *PTEN* mutations are associated with a number of tumor predisposition syndromes knowns as PTEN Hamartoma Tumors Syndromes, (PHTS) whereby affected patients develop disorganized and hyperplastic cellular overgrowths known as hamartomas that affect various tissues including thyroid, breast, skin, and brain, and can present neurodevelopmental disorders [15]. In somatic cancers, such as endometrial, breast, prostate cancer, and glioblastoma, PTEN inactivation encompasses an array of mutations which include missense and nonsense mutations, mono or bi-allelic deletion of the genomic locus or silencing through promoter methylation, and also targeting by oncogenic microRNAs [9,10].

Initial structural and functional studies in the late nineties highlighted how, despite the predicted role as a novel protein tyrosine phosphatase, PTEN catalytic activity presented a surprising high affinity toward phospho-lipid substrates with the second messenger PIP3 identified as the candidate of choice [16,17,18]. Thereafter, a number of studies confirmed that reductions in PTEN levels, or PTEN activity, not only induced PIP3 accumulation but also associated with activation of the proto-oncogene AKT, thus, establishing a key connection between a novel tumor suppressor and a functional target, the PI3K pathway [19].

The role of PTEN as a key tumor suppressor has been demonstrated and validated in multiple animal models and in vitro settings. In mice, constitutive *Pten* inactivation through either mono-allelic genomic loss or heterozygous expression of loss-of-function Pten mutations leads to tumorigenesis in multiple epithelial tissues including the mammary gland, prostate, thyroid, and adrenal glands [11,12,13,20,21]. In addition, conditional *Pten* inactivation through Cre–Lox systems confirmed that disruption of Pten function is tumor promoting in a cell autonomous fashion and that across the different tissues, the mammary gland is exquisitely sensitive to variations in the levels of this essential tumor suppressor [22,23,24,25]. Consistent with this, induced systemic *Pten* overexpression in the mouse triggers a tumor suppressive and cancer protective state through healthy metabolism, which indicates that pharmacologic strategies able to increase levels or expression or activity of wild-type PTEN should be exploited as novel treatment modalities for cancer prevention and therapy [26].

Pten inactivation frequently correlates with an active status of the PI3K pathway commonly measured by monitoring the levels of AKT phosphorylation which also suggests that AKT may be an important PTEN functional target [5]. In agreement with this, tissue-specific AKT deletion was shown to rescue the *Pten*-loss driven tumorigenesis in multiple tissues, particularly effective in endometrium and prostatic gland and interestingly, this occurred with distinct contribution of the various AKT isoforms [27,28]. Notably, despite the high degree of structural homology, AKT1, AKT2, and AKT3 present distinct expression patterns, unique post-translational modifications and display diverse intracellular localizations suggesting that mechanisms regulating expression profiles and function of these distinct AKT isoforms can affect in various, and sometimes opposite ways, the PTEN loss-driven tumorigenesis and also add a novel layer of complexity to our understanding of the PI3K signaling network [5,29,30].

### 2.2. PIP3 is More than AKT

PI3K activation or loss of PTEN equally leads to accumulation of the second messenger PIP3. While AKT has been demonstrated to execute many of the PIP3 biological functions, recent reports have also highlighted how PIP3 does not necessarily equals AKT activation but that additional context-dependent functional targets can better mediate the signal of this critical PI3K product [31,32].

**Figure 1:**
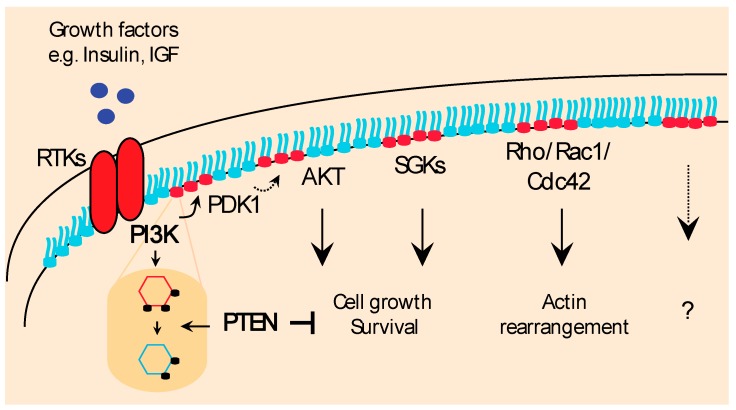
The phosphatase and tensin homolog deleted on Chromosome 10- phosphatidylinositol 3-kinase (PTEN–PI3K) axis modulates activation of multiple pro-survival signal transduction pathways. In physiological conditions, growth factors stimulate PI3K which once active phosphorylates the phospholipid substrate phosphatidylinositol (4,5)-bisphosphate (PIP2) to generate the second messenger phosphatidylinositol (3,4,5)-trisphosphate (PIP3). PIP3 recruits and activates a number of functional targets, such as phosphoinositide-dependent protein kinase-1 (PDK1), AKT isoforms, the serum and glucocorticoids-regulated kinases, (SGKs) signaling pathways and the Rho family of GTPases (Rho/Rac1/Cdc42), and promotes a plethora of biological effects to sustain cell growth, proliferation and cytoskeleton modification. The lipid phosphatase PTEN converts PIP3 to PIP2 and terminates propagation of the growth signal to maintain normal cellular and tissue homeostasis. RTK: Receptor Tyrosine Kinases; IGF: Insulin-like Growth Factors

In an important paper from the Garraway lab, *PIK3CA* mutations or *PTEN* inactivation were shown to differentially correlate with AKT phosphorylation levels in a number of cancer cell lines and tumor samples [32]. Importantly, however, loss of PTEN better correlated with high levels of AKT phosphorylation than did *PIK3CA* mutations. Moreover, mutant PI3K samples with low levels of AKT phosphorylation were instead associated with activation of the PDK1–SGK3 signaling pathway whose activation supported cell viability more efficiently than AKT [32]. Additionally, independent studies have shown that PIP3-binding proteins, such as the PIP3-dependent Rac exchange factor 1 (P-REX) and the Rho/Rac/Cdc24 family members, are all implicated in supporting metabolic reprogramming, cytoskeleton remodeling, cell growth, and cell division in an AKT-independent fashion [33,34]. Thus, PIP3 can activate a number of parallel signaling pathways that independently function to promote growth and survival and are, therefore, implicated in the pathogenesis of cancer, beyond AKT. This can have important therapeutic implication particularly for PIK3CA and/or PTEN mutant cancers, Figure 1.

## 3. The Proto-Oncogene PI3K and PTEN

### 3.1. *PIK3CA* Mutations and Functions Vis a Vis PTEN Regulation

The family of lipid kinases PI3Ks encompasses three classes of enzymes and eight different isoforms which display overlapping as well as distinct biological functions [1]. Because of their implication to cancer, here we will focus mostly on the role of class IA PI3Ks and their cross-talk with the tumor suppressor PTEN. For details on functions and regulation of additional classes of PI3Ks we refer the reader to accompanying reviews within this book.

Class IA PI3Ks phosphorylates the 3′-hydroxyl position of the PI(4,5)P2 to generate the second messenger PIP3. Of the three isoforms belonging to this class of kinases, *PIK3CA* is the only gene ubiquitously expressed and frequently mutated in human cancer [2,35]. *PIK3CA* amplification or clustering of somatic mutations in either the helical domain (e.g., G545L) or the kinase domain of the p110α subunit (e.g., H1047R) occurs in up to 30% of colon, brain, breast, and gastric cancers [2]. In vitro and structural studies have suggested that these cancer-associated mutations affect p110α activity through two possible mechanisms [36]. They can either induce a release from the p85 inhibitory effect, thus conferring p110α an increased baseline catalytic activity, as observed with mutations in the helical domain; or they can impose a conformational change in the activation loop, as described for mutations in the H1047 site [37,38]. Regardless, the final outcome is an increased p110α activation which promotes tumorigenesis and multipotency [39,40,41].

Upon growth factors stimulation, the p85:p110 heterodimer is recruited to the membrane where binding of the p85 regulatory subunit to phosphorylated tyrosine residues on RTKs or adaptor molecules derepresses the inhibitory switch on p110 and allows p110 activation and PIP3 production [36]. Accumulation of PIP3 is tightly controlled by PTEN lipid phosphatase activity, and recent reports have described a direct binding between PTEN and p85α, one of the five p85 isoforms expressed in mammals [42]. The proposed model suggests that in a homodimeric conformation, p85α, is able to directly bind an unphosphorylated PTEN and enhance PTEN stability, membrane recruitment and lipid phosphatase activity toward PIP3 [43,44]. Consequently, this would establish a negative feedback loop whereby mechanisms promoting PIP3 generation, mediated by p85α, can also initiate a protective response that terminates its function by favouring recruitment and engagement of PTEN. Interestingly, we and others have demonstrated that PTEN exists as an unphosphorylated homodimer and that in this active conformation PTEN dephosphorylates PIP3 [20,45]. Thus, a fine equilibrium of homo- (PTEN:PTEN and p85α:p85α) and hetero-dimers (p85α:p110 and p85α:PTEN) characterizes the formation of a functional molecular complex, also known as the PTEN associated complex (PAC), that as a consequence of diverse post-translational events, such as phosphorylation and ubiquitination, assembles at the membrane and efficiently regulates PIP3 production and hydrolysis. Future studies and structural analysis will be required to validate these findings in multiple experimental settings.

### 3.2. p110 Isoforms, Targeted Therapies and PTEN

Cancer-associated *PIK3CA* mutations occur frequently in breast cancer and are oncogenic in mammary epithelial cells and glands [41,46]. In vitro studies and a number of independently generated in vivo mouse models have shown that conditional expression of mutant PI3K, often the hotspot H1047R mutation induced by various mammary gland-active Cre (e.g., mouse mammary tumor virus (MMTV)-Cre, whey acidic protein, (WAP)-Cre), promotes late onset mammary tumorigenesis (i.e., over 13 months) which is accelerated by multiple rounds of pregnancies [41,47]. Because of the frequency of *PIK3CA* mutations and their oncogenic role in vivo, the lipid kinase PI3K has become a highly sought-after target for the development of novel anticancer drugs [48]. Multiple small molecules have been developed over the years with the capacity to either specifically target single p110 isoforms (isoform-specific inhibitors) or to recognize multiple, if not all p110 isoforms through the generation of isoform-sparing inhibitors or pan-PI3K inhibitors [4]. Efficient inhibition of all p110 isoforms, although desirable for cancer suppression, has however presented a number of counterproductive effects mostly associated with dose-limiting toxicities that have hindered the optimal therapeutic response to these drugs in the clinic [49]. Targeting single PI3K isoforms has, therefore, emerged as a safer therapeutic option because isoform-specific PI3K inhibitors are better tolerated by patients and can also be more easily combined with additional targeted therapies as is currently being tested with hormone therapies and cell cycle checkpoint inhibitors for the treatment of multiple types of breast cancer [50]. Beyond toxicity, pre-clinical and clinical studies have also shown that a key challenge for the identification of optimal therapeutic windows for PI3K inhibitors is a better patient stratification and the identification of biomarkers of response that can predict sensitivity to treatment [51]. To this end, *PIK3CA* mutant patients have been shown to display better response rates to PI3K inhibitors than patients with wild-type *PIK3CA*; similarly, activating AKT mutations, such as the E17K, can predict a better response to AKT inhibitors [52]. Thus, is not just the activation of the PI3K pathway per se but rather the specific molecular mechanisms promoting the oncogenic signal that can help develop tailored treatments and more efficacious therapies for cancer management [53].

**Figure 2:**
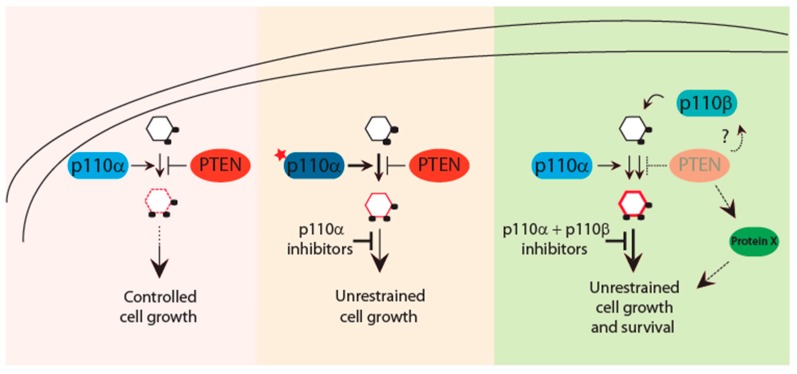
The PTEN-PI3K axis dictates response to PI3K-directed therapies. Finely regulated PIP3 levels are under the control of the PI3K–PTEN axis. Acquisition of *PIK3CA* mutations tilts the physiologic balance to overcome PTEN inhibition and promote tumorigenesis. *PIK3CA* mutant cancers are sensitive and better respond to isoform-specific PI3K inhibitors. Loss of PTEN function has been associated with activation of multiple p110 isoforms that act with tissue-specific dependencies. Thus, PTEN-deficient tumors may require inhibition of more than one p110 isoform for effective therapies. Further, multiple PTEN-protein targets have been identified which can contribute to the PTEN-driven tumorigenesis. These substrates together with PI3K inhibitors can provide a better therapeutic option for PTEN mutant cancers.

Nevertheless, the efficacy of PI3K inhibitors in the clinic cannot prescind from the functional status of PTEN. Indeed, a clinical trial testing efficacy of the p110α-isoform-specific inhibitor alpelisib, found that six breast cancer patients harboring *PIK3CA* mutations displayed initial positive response to treatment until selective pressure promoted acquisition of novel mutations in the tumor suppressor *PTEN* which associated with metastatic growth and eventually led to treatment failure [54]. Mechanistically, complete *PTEN* loss was found to overcome drug inhibition by promoting engagement of the p110β isoform, hence, inducing a compensatory reactivation of the PI3K pathway. PTEN regulation of the p110 isoforms switch has been observed and reported in multiple types of cancer, and in prostate cancer, p110β activation has been shown to primarily mediate *PTEN*-loss driven tumorigenesis [55,56,57,58]. Moreover, a few studies have also shown that in cancer, *PIK3CA* and *PTEN* mutations are not necessarily mutually exclusive but that their concomitant alterations can have biological effects and therapeutic implication [59,60,61]. Thus, a number of critical questions remain to be addressed, such as: what are the molecular mechanisms that select for one versus the other p110 isoforms in a context of PTEN deficiency? And in a setting of combinatorial PI3K and PTEN mutations, are the distinct p110 isoforms all functionally relevant? And, are there additional p110-independent signaling pathways emanating the oncogenic signal driven by PTEN loss? To date, there is no evidence of a direct binding between PTEN and any of the p110 isoforms that could help address these questions and even though PTEN has been shown to bind p85α, it is not known whether this interaction is conserved across the remaining p85 isoforms. Further, PTEN itself executes a number of tumor suppressive functions which can be mediated by lipid phosphatase-dependent and independent activities and that have been shown to play important role in cancer suppression and resistance to therapies, as we detail below (Figure 2) [62].

## 4. PTEN Beyond PI3K

### 4.1. PIP3-Independent PTEN Functions

Despite the many shared functional targets, loss of PTEN in preclinical settings has not demonstrated as clear an association in terms of response to PI3K inhibitors as observed with mutant PI3K [52]. In cell lines and xenograft studies, Tanaka et al. found that *PIK3CA* mutant cells consistently displayed high sensitivity to the class I PI3Ks inhibitor CH5132799, whereas PTEN loss was associated with positive as well as negative response to the drug in vitro and in vivo [63]. Similarly, in a panel of 18 breast cancer cell lines tested in response to the dual PI3K and mTOR inhibitor BEZ235, it was found that the presence of *PIK3CA* mutations and *HER2* amplification strongly associated with growth inhibition and apoptosis in response to the drug, whereas mutant PTEN cells were seemingly protected and displayed high phosphorylation levels of the mTORC1 target Ribosomal Protein S6 (RPS6) by the ERK pathway [64]. Further, in a mouse model of prostate cancer, we found that *Pten* loss is accompanied by a profound suppression of mitogen-activated protein kinase (MAPK) signaling. However, *Pten* loss potently cooperates with inactivation of the tumor suppressor *Pml* that promotes increased lipogenesis and progression to metastasis specifically through reactivation of the MAPK pathway [65]. Thus, while PTEN-deficient tumors can rely on multiple p110 isoforms for growth and survival, in vivo models and in vitro studies of drug sensitivity in response to Pan-PI3K or dual PI3K inhibitors should take into accounts signaling consequences that may not be directly dependent on PIP3, and that are triggered by accompanying mutations.

### 4.2. PTEN Lipid and Protein Phosphatase Activity Side-by-Side

Early studies in 1997 identified PTEN as a novel tumor suppressor belonging to the superfamily of tyrosine receptor phosphatases and, more specifically, PTEN was described as a dual specificity protein-phosphatase with activity towards phospho-serine and threonine and phospho-tyrosine peptides [66]. Ever since, multiple phospho-proteins have been identified and proposed as direct PTEN targets, but the contribution of these functional targets to the well-established PTEN biologic function has remained contentious, reviewed in [9].

In trying to address this issue through an in vivo approach, we and others generated *Pten* knock-in (KI) mice harboring loss-of-function PTEN mutations known to differentially affect the distinct PTEN catalytic activities [20,21]. We selected the cancer-associated Pten G129E mutation which was shown to affect PTEN lipid function while leaving PTEN protein phosphatase activity intact, and in a second model, we targeted the *Pten* exon 5 to generate mice expressing the Pten C124S mutation which lack lipid and protein phosphatase activity and renders Pten phosphatase dead [66]. The Leone laboratory chose to model the Pten G129E mutation and the Pten C124R mutation with similar effect on Pten catalytic activity [21]. In these studies, we found that homozygous expression of both mutations was lethal in the uterus and homozygous KI mice died at similar stage during embryonic development, thus, suggesting that Pten activity towards phospho-lipid substrates is essential for proper embryogenesis. In adulthood, heterozygous expression of either Pten mutations promoted tumor development in a number of tissues with no obvious difference between the two Pten KI models in terms of tumor onset and disease progression. However, through careful analysis, we also discovered that Pten C124S/+ mice presented twice as many adenomas of the pituitary gland than PtenG129E/+ mice and that Pten C124S/+ male mice developed complete testicular atrophy by 10 months of age which was not found in Pten G129E/+ mice. Further, loss of Pten lipid function induced formation of pre-malignant hepatic fatty nodules which were rarely found in Pten C124S/+ mice suggesting that loss of PTEN protein phosphatase activity could somehow rescue the liver phenotype [20]. Thus, through these studies we conclude that although the PTEN lipid phosphatase activity is responsible for many of the PTEN loss-driven phenotypes, consistent with the critical role of PI3K and PIP3, but that in a tissue-specific manner, or under particular physiologic conditions, such as stress, aging, or cell regeneration, PTEN protein phosphatase activity becomes more apparent, and in that, it modulates mechanisms of survival and growth. Consistent with this hypothesis, Zhang et al. reported that loss of PTEN contributed to resistance to Trastuzumab treatment in erb-b2 receptor tyrosine kinase 2 (ERBB2)-overexpressing breast cancer cell lines and xenograft models by directly dephosphorylating and activating the proto-oncogene and nonreceptor tyrosine kinase (c-SRC) on tyrosine 416 [67]. Thus, upon drug-targeting, PTEN lipid and protein phosphatase activities both impact response to treatments and synergistically act to coordinate cell survival. This also indicates that the functional relevance of PTEN protein phosphatase activity may be revealed under specific biological conditions and that validated PTEN protein targets should also be taken into consideration, in addition to PI3K inhibitors, for the treatment of PTEN-driven diseases including cancer. Generation of Pten KI mice with specific loss of PTEN protein phosphatase activity only and intact lipid function as observed with the PTEN Y138L mutation will further help to thoroughly address this issue [68].

## 5. Conclusions

In this review, we have summarized key functions and regulatory mechanisms associated with the PTEN–PI3K axis in cancer. We have dissected the functional crosstalk of PTEN and PI3K with additional components of the PI3K pathway and highlighted the important role of established as well as novel mediators of the pro-survival PIP3 signal. Further, we have integrated the conventional role of PTEN as a master regulator of PIP3 levels and proposed a novel concept in favour of a PIP3-independent role of the PTEN catalytic functions in response to therapies and beyond, and hope that through future studies these, as well as additional theories and hypotheses, will lead us to a better and more comprehensive understanding of the PI3K signaling network towards effective combinatorial treatments for cancer.
